# Automated classification of optical coherence tomography images of human atrial tissue

**DOI:** 10.1117/1.JBO.21.10.101407

**Published:** 2016-02-29

**Authors:** Yu Gan, David Tsay, Syed B. Amir, Charles C. Marboe, Christine P. Hendon

**Affiliations:** a Columbia University, Department of Electrical Engineering, 500 West 120th Street, New York, New York 10027, United States; b Columbia NY Presbyterian Hospital, 630 West 168th Street, New York, New York 10032, United States; c Columbia University Medical Center, 630 West 168th Street, New York, New York 10032, United States

**Keywords:** optical coherence tomography, image processing, classification, cardiac imaging

## Abstract

Tissue composition of the atria plays a critical role in the pathology of cardiovascular disease, tissue remodeling, and arrhythmogenic substrates. Optical coherence tomography (OCT) has the ability to capture the tissue composition information of the human atria. In this study, we developed a region-based automated method to classify tissue compositions within human atria samples within OCT images. We segmented regional information without prior information about the tissue architecture and subsequently extracted features within each segmented region. A relevance vector machine model was used to perform automated classification. Segmentation of human atrial *ex vivo* datasets was correlated with trichrome histology and our classification algorithm had an average accuracy of 80.41% for identifying adipose, myocardium, fibrotic myocardium, and collagen tissue compositions.

## 1 Introduction

Cardiovascular disease (CVD) is the leading cause of mortality and morbidity in the United States.^1^ An important factor in the pathophysiology of CVD is the composition and remodeling of the myocardium. Myocardial tissue includes muscle, adipose tissue, collagen fibers, and fibrotic myocardium, and the relative percentage of each varies by chamber and with the progression of the disease. Myofiber disarray can impair electrical conduction and result in arrhythmias or hypertrophic cardiomyopathy.^2^ The presence of adipose within the myocardium provides a high indication of arrhythmogenic cardiomyopathy^3^ and thickened layers of collagen fibers imply severe myocardial scar.^4^ The diffusion of myocardial fibrosis, a fundamental process in the remodeling of cardiomyopathy, is postulated to cause increased cardiac stiffness and poor clinical outcomes.^5^ Due to the importance of myocardial tissue composition on normal heart function and CVD, characterization of myocardial tissue can facilitate the evaluation of tissue remodeling, identification of arrhythmogenic substrates, and diagnosis of CVD.

In the past decades, medical imaging modalities including ultrasound,^6^^–^^8^ multidetector computed tomography,^9^^,^^10^ and magnetic resonance imaging have been used to characterize cardiac tissue compositions such as collagen region during myocardial infarction,^6^^,^^9^^,^^10^ adipose tissue,^8^^,^^11^ or organization of myofibers within myocardium.^7^^,^^12^ However, the abovementioned modalities suffer from either a low image resolution or a long data acquisition time. Optical coherence tomography (OCT) has been demonstrated to have the ability to image biological tissue at a fast rate with a high resolution (∼10  μm) with a 2 mm imaging range^13^^,^^14^ in the axial direction. Previous research efforts demonstrated that OCT can image important features within the heart^15^ such as the purkinjie network,^16^ atrial ventricular nodes,^17^^,^^18^ sinoatrial nodes,^19^ and myofiber organization.^20^^–^^23^ Given that the wall thickness in the human atria ranges from 2 to 5 mm,^24^ OCT has the ability to visualize a large percentage of the human atrial wall. There is a great potential to classify tissue compositions within human atria via OCT imaging. However, manual interpretation of OCT images is time consuming and not applicable for analysis on large three-dimensional (3-D) volumetric datasets. Therefore, automated identification of tissue composition in human atria from OCT images is greatly needed.

In this study, we present an image-processing algorithm to automate the classification of tissue compositions within atrial OCT images. Layer structures of multiple tissue compositions are automatically segmented using graph searching without any prior information. Within each layer, optical properties, statistical measurements, and texture features are extracted. Features are subsequently used to build a statistical classification model to distinguish tissue compositions of dense collagen, loose collagen, adipose tissue, normal myocardium, and fibrotic myocardium. Our work enables, to the best of our knowledge, the first automated classification of myocardial tissue compositions from human atrial OCT images.

## 2 Methods

### 2.1 Tissue Collection

Human hearts (n=15) were obtained under two approved protocols from the National Disease Research Interchange (NDRI). The inclusion criteria for the first NDRI protocol are based on the following diagnosis: end stage heart failure, cardiomyopathy, coronary heart disease, or myocardial infarction. The second protocol requests normal hearts. Fresh tissue samples were shipped submerged in ice-cold phosphate-buffered saline and received within 48 h of donor death. Detailed characteristics of the donor hearts within this study are listed in Table 1.

**Table 1:** Clinical characteristics of heart donors in dataset.

Characteristic	Value
N	15
Demographic profile	
Age in yrs, median (interquartile range)	66.0 (62.25 to 69.75)
Male, n (%)	5 (33.3)
BMI, median (interquartile range)	29.25 (24.3 to 34.2)
Medical history, n (%)	
Diabetes	6 (40.0)
Hypertension	2 (13.3)
Heart failure	2 (13.3)
Cardiomyopathy	1 (13.3)
Cause of death, n(%)	
Cardiac arrest	3 (20.0)
Cardiopulmonary arrest	3 (20.0)
Respiratory arrest	1 (6.7)
Respiratory failure	2 (13.3)
Chronic obstructive pulmonary disease	4 (26.7)
Congestive heart failure	2 (13.3)
Complete characteristics were not available for all donors	

### 2.2 Image Protocol

All samples were imaged *ex vivo*, using a spectral domain OCT system, Telesto I (Thorlabs GmbH, Germany). The system is an InGaAs-based system with its source centered at 1325 nm and a bandwidth of 150 nm. The axial and lateral resolutions are 6.5 and 15  μm in air, respectively. All datasets were acquired at 28 kHz. In our experiments, each volume consists of 800×800×512  voxels, corresponding to a tissue volume of 4  mm×4  mm×2.51  mm (in air). To extract the raw OCT data, the postprocessing algorithm, including λ to k space interpolation, windowing, and Fourier transform, was implemented using MATLAB 2014b (Mathworks, Inc., Massachusetts).

### 2.3 Histological Evaluation

Sections of tissue from the imaging field of view were processed for histopathology. Samples were sectioned parallel to the direction of the B-scans. Sample pieces were cut corresponding to the size of the OCT volume, fixed in formalin for ∼24  h, and then placed in ethanol (20%) for ∼24  h. After fixation, samples were stained with Masson Trichrome. For 33.3% of the samples, histology was taken every 2 mm to ensure multiple matches between histology and the OCT image set. For validation, two investigators, blind to the automated results, segmented and classified the images based on the histology. One-way analysis of variance with Tukey multiple comparison test tissue were performed to detect differences between the tissue compositions for each of the extracted features. A p-value of 0.05 was considered statistically significant. All statistical analysis was conducted with the software package Prism 6.03 2013 (GraphPad Software, Inc, California).

### 2.4 Algorithm Flow

**Fig. 1:**
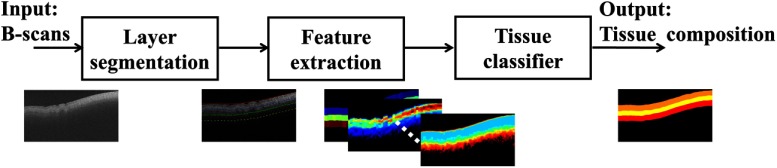
Flowchart of the automated algorithm for tissue classification of OCT images of human atrial tissues. B-scans from the OCT dataset were automatically segmentated into layers. Features were extracted for each layer and input to a classifier. The final output was the tissue composition.

To identify tissue compositions within OCT images, we present a region-based classification method. A schematic of the workflow for the analysis of two-dimensional (2-D) images is shown in Fig. 1. The algorithm consists of three steps: layer segmentation, feature extraction, and tissue classification. In each B-scan, OCT images were first segmented into multiple regions. Within the segmented region, features such as optical properties, texture analysis, and high order statistical moments were extracted. The features were inputs to a tissue classifier whose output was the tissue type for the region.

### 2.5 Layer Segmentation

**Fig. 2:**
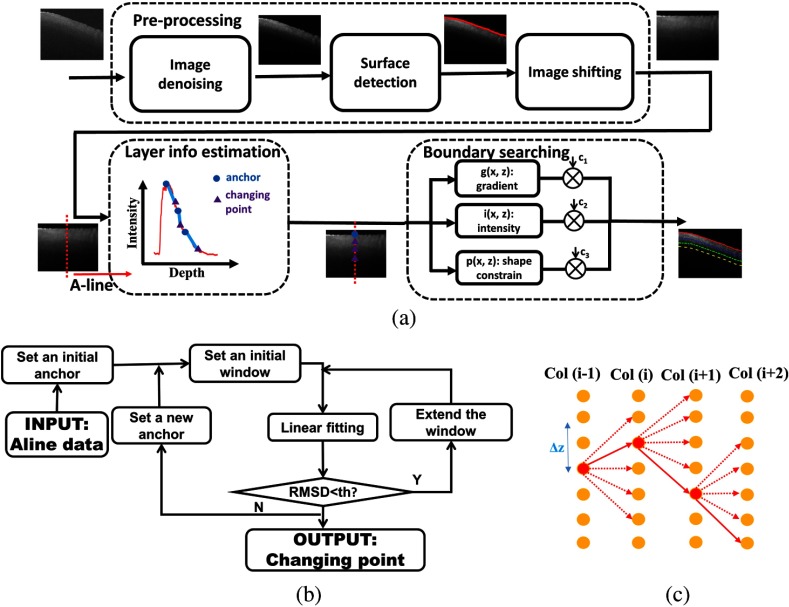
Layer segmentation algorithm. (a) Flowchart of layer segmentation algorithm; (b) flowchart for estimation of layer information within B-scan image; (c) schematic of boundary searching algorithm. Layer information, including number of layers and changing point of tissue structure were estimated in (b). Pixels at the boundary of each layer were searched from column to column within a range of Δz pixels over depth in (c).

For the first step, we divided OCT images into multiple layers through segmentation for future feature extraction and classification. Compared with existing segmentation methods, segmenting OCT images of atrial tissue is challenging. Within prior work, layer boundaries were automatically determined by minimizing a cost function^25^ or building a minimum weight graph.^26^ The weighting scheme, and searching order are determined by prior knowledge of the layer structure,^27^^,^^28^ such as empirical thickness measurements and knowledge of bright-to-dark transition patterns between two layers. Unfortunately, neither empirical thickness measurement or transition patterns is consistent for atrial tissue. In the atrium, layer thicknesses and tissue composition vary within a normal heart depending on the region that is imaged. Furthermore, the layer thicknesses and tissue composition change with the progression of the disease. Therefore, the first step of our algorithm is layer segmentation, which includes preprocessing, layer information estimation, and boundary searching. A detailed flowchart of the layer segmentation steps is shown in Fig. 2(a). The preprocessing step improves the image quality through denoising and flattens the image to reduce the boundary searching range. The layer information estimation step determines the number of tissue compositions and identifies starting points for boundary searching. The image is segmented after boundary searching.

#### 2.5.1 Preprocessing

The preprocessing procedure includes image denoising and flattening. Given that OCT images are generally corrupted by speckle noise,^29^ we used a block matching 3-D (BM3-D)^30^^,^^31^ method to denoise OCT images and enhance the boundaries. Briefly, for the BM3-D algorithm we divide the original OCT image into multiple blocks and denoise similar blocks. The BM3D method exploits the sparsity of structural information and is thus considered to be a good tool to denoise speckle noise and enhance boundary information. To reduce the searching range and maintain a smooth searching shape, we flattened and shifted the filtered image based on the tissue surface. To flatten/shift images in a fast manner, we undersampled the original image. In cardiac tissue, the most hyper reflective surface is in the endocardium. Within a downsampled A-line, we thus estimated the location of the maximum pixel value as the axial location of the surface. Then the image was shifted based on an interpolation of the axial location of the maximum value-pixels within the downsampled image.

#### 2.5.2 Layer information estimation

After image denoising and flattening, we estimated the layer information within each OCT image. The layer information consisted of the number of layers and the initial points for boundary searching between layers. To count the number of layers, we analyzed averaged A-lines in the OCT image. Due to the positioning of the sample and uneven sample surface, A-Lines around the center of the B-scan had better image quality than the regions toward the left and right edges of the image. To ensure an accurate estimation, five A-Lines were selected around the center of the image. The A-Lines were 200  μm away from each other. For each A-Line, 20 A-lines were averaged. In each averaged A-line, the intensity curve was linearly fitted using a sliding window. The algorithm flow of linear fitting is shown in Fig. 2(b). We first set the location of the maximum pixel value as the first anchor. Within a window, the intensity was linearly fitted. We calculated the root mean square deviation (RMSD) error as following:

RMSD=∑i=1N(y^i−yi)2/N, (1)

where y^i is the linearly fitted estimation and $y_{i}$ is the original intensity value. If the RMSD is below a threshold, it is assumed that the window is still within the same layer and thus the original window is extended to cover more range. Otherwise, if the RMSD is higher than the threshold, this iteration of layer searching is completed and we record the end of the window as a changing point. In the next layer, we set a new anchor that is one initial window size away from the recorded changing point to start a new linear fit. Since the A-Line data is noisy, a fixed distance between the changing points and the new anchor is needed to ensure that we are analyzing a new layer rather than repeatedly searching in the previous layer. In this study, we empirically set the initial window size as 10 pixels and the window size is extended three pixels during each iteration. The estimation resulted in a piecewise linear function. The number of layers in the OCT image was defined by the number of linear pieces within the A-line. In our implementation, we deleted any two neighboring changing points that were too close (<30  pixels) and set a new changing point that locates in the middle of two deleted changing points. If the changing points were due to high standard deviation of the intensities within the pixels around the peak of the A-line, the changing point would be deleted as well. Each changing point of the piecewise linear function was considered as one of the candidates used as initial boundary points for boundary searching. Upon determining the number of layers and changing points in each segment, a voting system was used to globally determine the number of layers and corresponding initial boundary points.

#### 2.5.3 Boundary searching

The boundaries were searched from the center of the image and progressed outward to the left and right. The boundary search algorithm minimizes the following function:

$$E(f)=E_{\text{data}}(f)+E_{\text{smooth}}(f),$$ (2)

where f is the label of the estimated surface, $E_{\text{data}}(.)$ is the energy of each pixel, and $E_{\text{smooth}}(.)$ is the energy quantifying smoothness of the estimated surface. To minimize E(f), we set a cost function c(x,z) as following:

$$c(x,z)=c_{1}g(x,z)+c_{2}i(x,z)+c_{3}p(x,z),$$ (3)

where g(x,z) is the gradient in the axial direction; i(x,z) is the intensity; p(x,z) is a weight defining layer structure. The term $E_{\text{data}}(f)$ in Eq. (2) is represented by g(x,z) and i(x,z) and the term $E_{\text{smooth}}(f)$ in Eq. (2) is represented by p(x,z). In general, the highest value of c(x,z) in Eq. (3) corresponds to the lowest energy in Eq. (2). This means that pixels at the boundary will have a high gradient, a high intensity, and a high weight for the shape constraint. The smoothness, p(x,z), is determined by the changing points obtained from the layer information estimation step. Generally, for each column, p(x,z) is large when (x,z) is close to changing points and is small when the pixel is in the middle of two estimated changing points. Three factors are weighted by $c_{1}$, $c_{2}$, and $c_{3}$ with a relationship of $c_{1}+c_{2}+c_{3}=1$. A combination of the factors we empirically used was 0.56, 0.38, and 0.06 for $c_{1}$, $c_{2}$, and $c_{3}$, respectively. Multiple boundaries were searched with the assumption that each boundary intersected with one column once. For each layer, starting from the changing point (from the anchor for the first boundary), we searched the boundary from one column to another. The searching range was (−Δz,Δz) of the determined boundary point in the previous column. Figure 2(c) shows a schematic of the boundary searching algorithm, starting from Col(i−1). The cost of 2Δz pixels was examined and the pixel with the highest weight was considered to be the boundary of the layer within Col(i). We then estimated the boundary point for the next column. The searching algorithm was run in parallel for multilayers within an image.

### 2.6 Feature Extraction

Within each segmented region, we extracted features from the OCT images to study different patterns of tissue compositions. The extracted features can be divided into three categories: measured optical properties, statistical moments, and texture analysis.

#### 2.6.1 Measured optical properties

Optical property parameters that we studied were attenuation coefficients (${\mathrm{mm}}^{-1}$) and penetration depth (mm). Attenuation coefficient was measured based on the method mentioned in Ref. 32. Penetration depth was defined as the depth at which the intensity drops to 1/e of its original intensity^33^ when light first reaches the layer. Additionally, we calculated the distance between the centers of layers to the tissue surface.

#### 2.6.2 Statistical moments

We performed histogram equalization and median filtering on raw OCT data. Then we calculated the statistics of high order moments (skewness and kurtosis), on the intensities of the denoised image within the whole layer to analyze the distribution of intensity for various tissue types.

#### 2.6.3 Texture analysis

We encoded OCT images with texture on equalized and filtered OCT images. Texture feature number (TCN)^34^ is assigned to each pixel. In TCN, the local feature of each pixel is represented by the intensity change of its eight neighboring pixels. We analyzed the statistics of the TCN number within each layer. In particular, we calculated the coarseness and homogeneity from the histogram of the TCN. We also quantified the mean and standard deviation from the default texture analysis tool in MATLAB, such as range filter and std filter, within each region. We also quantified entropy within the region. In addition, we constructed gray level cooccurrence matrices (GLCM)^35^ to extract more additional texture features. Specifically, contrast, energy, and correlation were computed by setting the number of levels to 16.

**Fig. 3:**
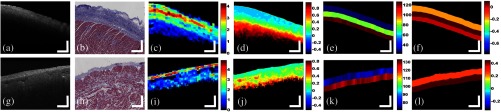
Example tissue images obtained from OCT, histology, and parametric images. (a) Original OCT image collected from the left atrium. (b) Trichrome histology of the same sample used in (a). (c) Attenuation coefficient map obtained from (a) (unit: ${\mathrm{mm}}^{-1}$). (d) Entropy map obtained from (a) (unit: 1). (e) The layer depth map obtained from (a) (unit: pixel). (f) The skewness map obtained from (a) (unit:1). (g) Original OCT image collected from the right atria. (h) Trichrome histology of the same sample used in (g). (i) Attenuation coefficient map obtained from (g) (unit: ${\mathrm{mm}}^{-1}$). (j) Entropy map obtained from (g) (unit: 1). (k) The layer depth map obtained from (g) (unit: pixel). (l) The skewness map obtained from (g) (unit:1). For different tissue composition in OCT images, they are showing different signatures in attenuation coefficients, entropy map, layer depth, and skewness. Scale bar: 500 um.

Representative parametric images obtained from left and right atrial samples are shown in Fig. 3, where we presented typical pixel-based, A-Line-based, and layer-based features. From pixel-based parametric images, such as attenuation coefficients and entropy in Figs. 3(c)–3(d) and Figs. 3(i)–3(j), large variations can be observed within a single layer. In A-line-based features, such as distance to the surface, there are smaller variations within each layer and the differences between tissue compositions can be well observed. For layer-based features, we performed texture analysis on pixels within the whole layer. Representatives are shown in Figs. 3(f) and 3(i). To simplify our model, we averaged the pixel-based and A-Line-based feature values within each layer. This resulted in a vector of features for each layer. The number of entries in the vector was the number of features. In this study, we extracted 16 features, listed in Table 2.

**Table 2:** List of features used in the classifier.

	Feature	Description
1	Attenuation coefficients (mean)	Mean value of attenuation coefficient
2	Attenuation coefficients (std)	Standard deviation of attenuation coefficient
3	Penetration depth (mean)	Mean value of penetration depth
4	Penetration depth (std)	Standard deviation of penetration depth
5	Std filter value (mean)	Mean value of standard deviation filtering results
6	Std filter value (std)	Standard deviation value of standard deviation filtering results
7	Range filter (mean)	Mean value of range filtering results
8	Range filter (std)	Standard deviation value of range filtering results
9	Entropy	Entropy of the pixel values
10	Coarseness (TCN)	Coarseness analysis of texture code number
11	Homogeneity (TCN)	Homogeneity feature of texture code number
12	Contrast (GLCM)	Contrast feature from GLCM
13	Energy (GLCM)	Energy feature from GLCM
14	Distance to surface	The distance between the center of the layer and the surface
15	Skewness	Skewness within the whole layer
16	Kurtosis	Kurtosis within the whole layer

### 2.7 Relevance Vector Machine Classifier

The relevance vector machine (RVM)^36^ was used to classify tissue compositions. For each feature vector x, the probability of the vector belonging to a specific tissue composition c was determined by the following equation:

$$p(c=1|w,x)=\sigma [\overset{B}{\underset{i=1}{\sum }}\omega_{i}\varphi_{i}(x)],$$ (4)

where w is the weight, ϕ(x) is a kernel function, σ(·) is a sigmoid function, and B is the number of vectors. A zero mean Gaussian prior is typically chosen for computational convenience for the weights.

$$p(w_{i}|\alpha_{i})=N(w_{i}|0,\alpha_{i}^{-1}).$$ (5)

The distribution is determined by the values of the hyperparameters $\alpha_{i}$. Given a dataset of input vectors with known tissue composition, $D=\{(x^{n},c^{n}),n=1,2,\ldots ,N\}$. The hyperparameter $\alpha_{i}$ can be set by maximizing the marginal likelihood

$$p(D|\alpha )=\int p(D|w)p(w|\alpha )dw=\int \overset{N}{\underset{n=1}{\prod }}p(c^{n}|x^{n},w){\left(\frac{\alpha }{2\pi }\right)}^{B/2}\exp(-\frac{\alpha }{2}w^{T}w)dw.$$ (6)

Here, we used the Gull–MacKay method to update $\alpha_{i}$.

New values for the weight vector w were estimated by calculating the derivative of the expectation of the weights ∇E[w] and the Hessian matrix of the weights H. Using a Newton update method, the new weights were estimated as

$$W^{\text{new}}=w-H^{-1}(\nabla E).$$ (7)

The classifier alternated in updating hyperparameters and weights. After obtaining a converged w, the training of the classifier terminated. Following the training, we estimated the probability of each unknown layer belonging to a specified tissue composition.

RVM is a Bayesian framework of the support vector machine (SVM), which is widely used in classification^37^^,^^38^ and segmentation.^39^ Compared with the SVM model, RVM obtains sparser solutions for weight vector w. This is done by adopting a nonGaussian prior for multiple hyperparameters $\alpha_{i}$, which only requires a limited number of weights w to be “active”. Once the values for the hyperparameter are optimized, most of the hyperparameters tend to move toward infinity. This results in most weights getting closer to zero, and becoming “irrelevant” in establishing a decision boundary. Only relevant weights are retained, which produced a significantly lower number of relevance vectors compared to SVM.

A leave-one-out experiment was conducted on the whole dataset. We used OCT images from 14 hearts as training data and used the images from the remaining one heart as testing data. The experiment was repeated such that images from any heart would be the testing dataset once.

### 2.8 Three-Dimensional Visualization

Given boundary information from the B-scans, we reconstructed the volumetric classification for myocardial tissue. Upon estimating the boundary from each B-scan, each layer can be roughly estimated. The detected boundary was arranged along the direction perpendicular to the B-scan. We further smoothed the estimated surface using a median filter and reconstructed the 3-D surface based on the smoothed plane. The estimated layer boundaries for each B-scan were modified accordingly. We then performed the tissue classification algorithm on each fine-tuned region in each B-scan. After performing the classification algorithm on each B-scan, the 3-D classification results were realigned. We overlaid the tissue composition with OCT volumetric data in an hue saturation value (HSV) scheme. In this study, tissue composition was encoded as hue; saturation and value are encoded as intensity. All 3-D results were visualized using the software package Amira 5.4.3 2012 (Zuse Institute Berlin, Germany).

## 3 Results

### 3.1 Segmentation Results

**Fig. 4:**
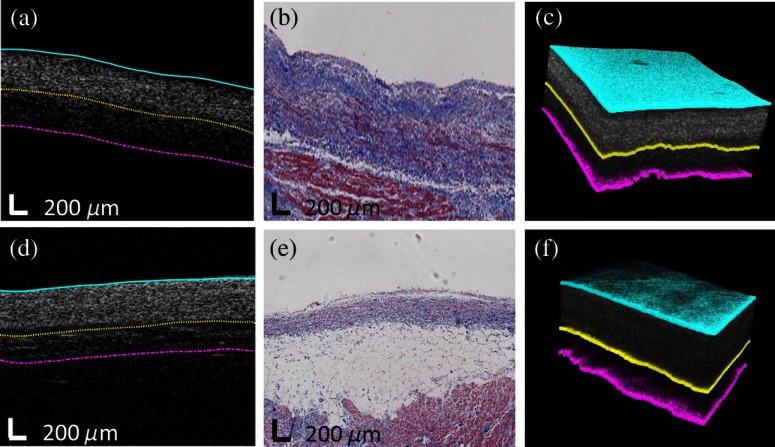
Segmentation results from human atria. (a) and (d) Original OCT images overlaid with automated segmentation result; (b) and (e) corresponding trichrome histology image; (c) and (e) 3-D segmentation results. The automated results in both 2-D and 3-D show great agreement with histology images.

Figure 4 shows two typical segmentation results of human atrial OCT volumes. In Figs. 4(a)–4(c), three layers are dense collagen (dark blue in histology), loose collagen (light blue in histology), and normal myocardium (red in histology) while in Fig. 4(d)–4(f), three layers are dense collagen (dark blue in histology), adipose tissue (white in histology), and normal myocardium (red in histology). In general, segmented boundaries matched the visible boundaries in the trichrome histology images. Moreover, we conducted quantitative comparisons between automated segmentations and manual segmentations from two observers for images from all 15 human hearts with corresponding histology. The results are listed in Table 3. The difference between automated segmentation and manual segmentation was 51.78±50.96  μm, which is comparable to results provided by the two investigators, 42.22±33.87  μm. To visualize boundaries in 3-D, multiple consecutive B-scans were segmented based on the filtered surface using the method in Sec. 2.8. The 3-D segmentation results are shown in Figs. 4(c) and 4(f).

**Table 3:** Comparison between automated segmentation and manual measurements from two observers.

	Automated versus observer 1		Automated versus observer 2		Observer 1 versus Observer 2	
	Mean (μm)	Std (μm)	Mean (μm)	Std (μm)	Mean (μm)	Std (μm)
RA	42.26	57.48	51.72	61.12	42.89	55.57
LA	64.14	45.13	44.27	46.36	42.22	33.87

### 3.2 Feature Analysis

**Fig. 5:**
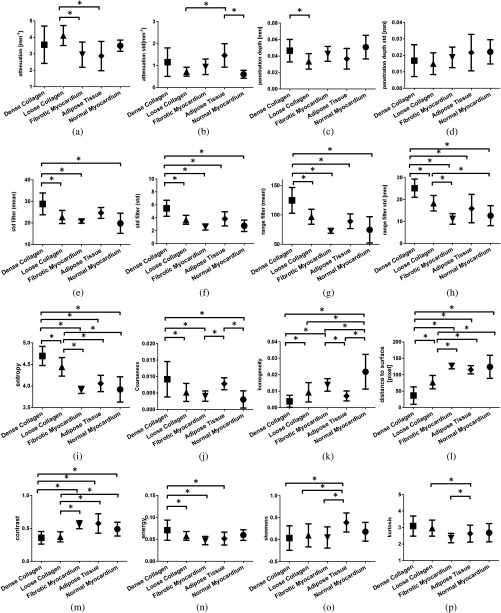
Statistical analysis of dense collagen, loose collagen, fibrotic myocardium, adipose tissue, and normal myocardium tissue in (a) mean of attenuation coefficient; (b) standard deviation of attenuation coefficient; (c) mean of penetration depth; (d) standard deviation of penetration depth; (e) mean of standard deviation filter; (f) standard deviation of standard deviation filter; (g) mean of range filter; (h) standard deviation of range filter; (i) entropy; (j) coarseness; (k) homogenity; (l) distance to surface; (m) contrast; (n) energy; (o) skewness; and (p) kurtosis. (p<0.05).

We performed statistical comparison of features for five tissue compositions: normal myocardium, loose collagen, adipose tissue, fibrotic myocardium, and dense collagen. Measurements from optical properties, statistics, and texture analysis were shown in Fig. 5. In general, we found that features from OCT images of endomyocardium had a strong correlation with tissue composition. We found that normal myocardial tissue was only significantly different from the all four tissue compositions in homogeneity (p<0.0074). Loose collagen was not significantly different from fibrotic in homogeneity and energy, but showed statistical differences in statistical moments (kurtosis, p<0.0325) and optical properties (attenuation coefficients p<0.0092). Within texture analysis, dense collagen was not significantly different from adipose tissue in homogeneity, but had significantly different values in energy (p<0.0144). Similar observations were found in the rest of features, thus all 16 features were used within the classification model.

### 3.3 Classification Results

**Fig. 6:**
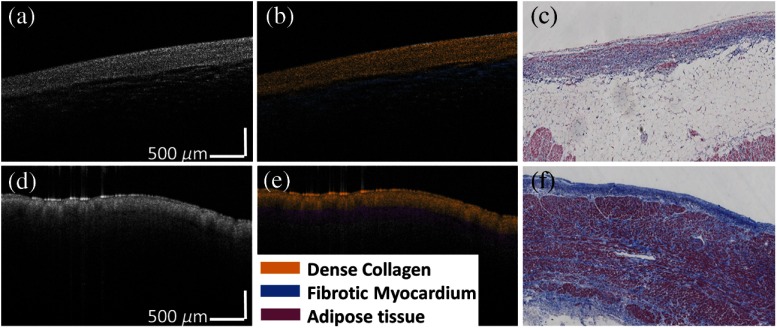
Two dimensional classification results from human atria. (a) and (d) Original OCT images; (b) and (e) color-coded automated classification image; (c) and (f) corresponding trichrome histology. The classification results show great agreement with histology images.

We performed classification experiments on 60 B-scans from 15 human hearts. Representative classification results are shown in Fig. 6. Similar to segmentation results, we performed comparison between automated classifications and trichrome histology and presented the comparison in Fig. 6(a)–6(f), of which 6(a) and 6(d) are raw OCT images, 6(b) and 6(e) are color-coded classifications, and 6(c) and 6(f) are histology images. The tissue compositions were color coded in HSV where hue encoded the tissue composition and saturation and value encoded intensity. Two layers are dense collagen (dark blue in histology) and adipose tissue (white in histology) in Figs. 6(a)–6(c) while dense collagen (dark blue in histology) and fibrotic myocardium (purple in histology) are shown in Figs. 6(d)–6(f). Both classification results agree with the trichrome histopathology.

**Fig. 7:**
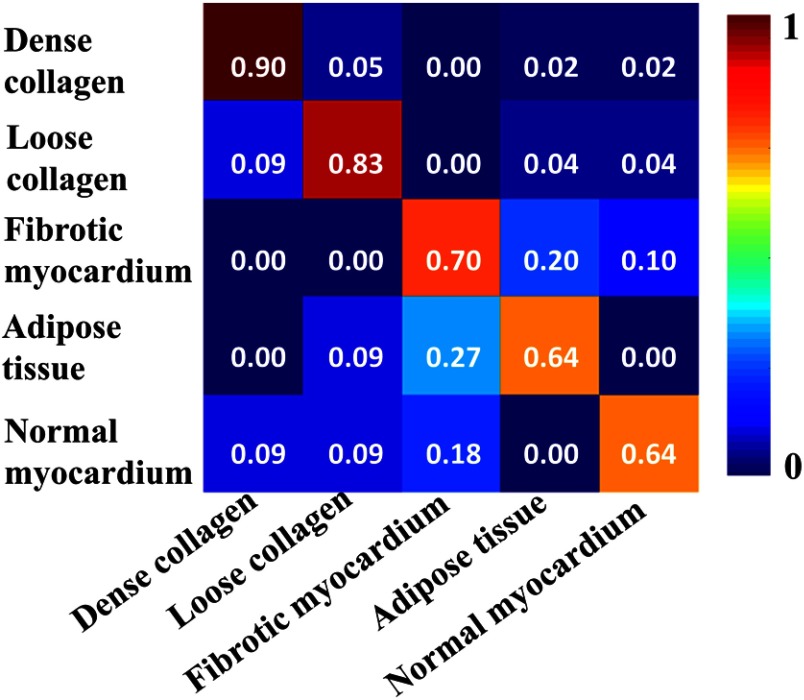
Confusion matrix of classification results.

A leave-one-out experiment was conducted on the whole dataset. We used OCT images from 14 hearts as training data and used the images from the remaining one heart as testing data. The experiment was repeated such that images from any heart will be the testing dataset once. To validate the accuracy, we compare the automated classification result with the tissue types shown in histology on a layer-wise basis. We obtained the confusion matrix to assess overall accuracy, Fig. 7. The final tissue classification estimation for the region was the class with the highest probability. Using this identification rule, we achieved an average accuracy of 80.41% for classifying the five tissue compositions.

**Fig. 8:**
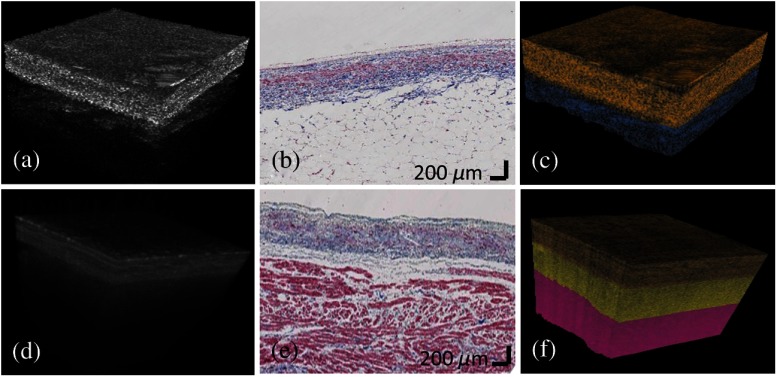
3-D classification results from human atria. (a) and (d) Original OCT volumes; (b) and (e) histology images; (c) and (f) color-coded classification results. Gold, yellow, red, and blue colors represent dense collagen, loose collagen, normal myocardium, and adipose tissue, respectively. The classification results delineated the layer structure and agreed with trichrome histology.

Representative 3-D classification results are shown in Fig. 8. The classified tissue compositions are overlaid on the original 3-D dataset using the HSV scheme. Gold, yellow, red, and blue hues represent dense collagen, loose collagen, normal myocardium, and adipose tissue, respectively. In Fig. 8(c), three tissue compositions of dense collagen, loose collagen, and normal myocardium are well classified and tissue compositions of dense collagen and adipose tissue are well specified in Fig. 8(f).

## 4 Discussion

In this paper, we presented an automated algorithm to characterize tissue compositions in human atrial tissue. To the best of our knowledge, it is the first work to automatically classify myocardial tissue using OCT. Our algorithm builds a probabilistic model to differentiate tissue compositions in OCT statistically, and achieved a high accuracy to identify tissue composition within human atrial samples *ex vivo*. Our method does not take into account prior information, and thus can have potential applications to other tissues including OCT images breast,^40^ oral,^41^ skin,^42^^,^^43^ and vascular tissues.^44^^,^^45^

Our classification algorithm had an 18% false positive rate of normal myocardium being misclassified as fibrotic myocardium. The tissue regions with small diffusion of fibrosis in myocardium contributed toward this high false positive rate. Moreover, the detection rate in adipose tissue was comparatively low because the adipose tissue is located at different depths, where the texture pattern varies. In Fig. 6(a), the adipose tissue is in a honeycomb structure when it is imaged in focus. When out of focus, the adipose tissue appears as an area of isolated dots.

In the preprocessing step, we detected the surface to shift image. The image shifting, widely used in the existing segmentation method,^25^^,^^26^ is necessary to determine the layer boundaries. We found that the difference between estimated layer boundaries and manual segmentation results were 203.61  μm in LA and 259.35  μm in RA if we process our algorithm without the flattening step. In the feature extraction step, our texture analysis is based on segmented atrial layers. We thus analyze the coarseness and homogeneity features in TCN. The index-wise TCN feature could be considered if we extend our algorithm to nonsegmented images.

One limitation of our study is that the time between a donor’s death and heart imaging is variable among all samples. We found that the viability of imaging is degraded when we compared OCT images from early shipped hearts with the late shipped hearts. We will consider the deliver time as a factor to normalize the OCT image in the future; moreover, the process of fixation during histology may result in shrinkage of the cardiac sample. It can impair the accuracy of correlation between histology and OCT image. In this study, we manually matched a small number of histology and the OCT images. Serial sections of histology can ensure that a variety of tissue features are observed. However, care will need to be taken to account for the fact that multiple samples are used in a training set from a single patient/chamber. Furthermore, imaging depth has an influence on image quality of OCT B-scans. Thus, to minimize the variations of imaging depth, we (i) maintain the distance between the tissue surface and the objective to be the same range in all our experimental scenarios and (ii) adjust image contrast based on the same thresholding and histogram equalization. With the translation towards *in vivo* use with a forward viewing catheter probe,^46^^,^^47^ contact imaging will be necessary and the sample location within the image will be constant.

In the future, we will need to implement a robust algorithm that take into account arbitrary shapes in which regions of tissue may present themselves. Although most of the endomyocardial tissue shows a layered structure, we notice there is a possibility that certain tissue compositions such as adipose tissue or a mixture of myocardium and blood vessels appear in a circular shape. It is possible that such a contour would not be not identified in the column-to-column search scheme. To overcome this drawback and make our algorithm more generalized, we will have a circular identification process and a refilling process. In particular, the Hough transform can be used to find circles and the snake algorithm to delineate the circular contour. Intensity values inside the circle will be refilled with the mean value of its neighboring pixels outside the circle. Then we will process layer segmentation and the tissue classification method as proposed in this study.

An important application of this work is for the development of improved cardiac models. We will incorporate atrial tissue characterization results into sample specific electrical physiology models of the human atria. A 3-D finite element model^48^ will be built based on the geometry of human atria. Tissue composition is important for understanding conduction properties and arrhythmia substrates.^18^ A second application is towards the goal of performing an “optical biopsy.” Most biopsy samples are around 1 to 2 mm^3^^,^^49^ similar to the size of volumetric data we usually acquired in the OCT system. We will extend our tissue classification algorithm to a model of ventricular myocardial characterization and data acquired with high resolution OCT systems.^50^ Our classification algorithm has the potential to guide the procedure of endomyocardial biopsy by avoiding areas with increased scar or/and performing optical biopsies where conventional biopsy is unsafe. Lastly, our classification method can be used to aid the treatment of atrial fibrillation by radiofrequency ablation (RFA).^24^^,^^51^^,^^52^ In particular, the identification of tissue composition can provide guidance for ablation operation and facilitate the evaluation of ablation performance.

## 5 Conclusion

We have developed an image processing tool to classify tissue compositions. We proposed an automated algorithm to segment layer structures within endomyocardium. Features including optical properties, high moment statistics, and texture analysis were extracted and compared. Based on extracted features, a probabilistic model was used to identify the tissue composition of segmentation. Segmentation results from human cardiac images agreed with histology. We achieved an accuracy of 80.41% in classification. Tissue composition information provided by this method can be used for a range of applications, to further understand the role of tissue composition on the electrical function of the heart, and translational applications for the monitoring and guidance of diagnostic and therapeutic interventions.
